# Mr. Huggan, on the Croup

**Published:** 1800-01

**Authors:** A. Huggan


### *To the Editors of the Medical and Phyfical Journal.*

GENTLEMEN, *Plymouth, 21ft October.*

I Refume the fubject of my former letter, with a few remarks upon Mr. FIELD's method of curing croup, as far as that is connected with it.*

Of this difeafe, there certainly are, as that gentleman juftly obferves, two diftinct fpecies; but that the nice difcrimination of them, with a view to their treatment, is abfolutely neceffary, is a circumftance to which I cannot fo readily agree, as both feem to have a proximate caufe, different only as to the part which it affects. The fpafmodic croup is occafioned by a fpafm of the mufcles of the glottis; the inflammatory kind confifts in a fpafm of the extreme arteries of the mucous membrane of the larynx and trachea.†

Mr. FIELD, like moft other writers on this difeafe, in his Plan of Cure, places his chief dependence upon venæfection. I need not fay, that I do not think this a very eligible practice, for I have my doubts whether it be fafe; even children do not bear the lofs of blood with impunity: indeed, it is to be feared, that to this very circumftance may be owing, in a great meafure, the frequent recurrence of the complaint, in thofe who have once been afflicted with it. I cannot help fufpecting farther,

* Medical and Phyfical Journal, Vol. II. page 146.

† Cullen's Firft Lines, &c. CCXL. CCCXVIII.

ther, that, by too copious a venæfection in children, we may be in danger of haftening what we are anxious to prevent—a fatal termination of the difeafe.

In a manufcript copy of the late Dr. Gregory's Lectures, I found a caution refpecting bleeding in children, even with leeches, as apt to bring on fits. Now, if the learned profeffor's admonition was the refult of experience, and a cafe which I myfelf once faw, leaves me little room to doubt it, what have we not to dread from taking away blood in a large ftream from infants?

The fymptoms of croup being fo very alarming, often threatening immediate death, demand the moft fpeedy as well as judicious exertions of the phyfician to combat them.

Former experience having taught him, that blood-letting has, in moft inftances, alleviated all the violent fymptoms of the difeafe; and thinking it unfafe to truft to any other remedy, however equally efficacious that may be, in producing the fame good effect, from the fear of its not being as quick in its operation, his own profeffional character, perhaps, being at ftake, in cafe of failure, from trying a mode oppofite to that which has received the fanction of the greateft names in the profeffion, he is therefore compelled, by a fort of neceffity, to have recourfe to bleeding, as the fpeedieft means of averting the prefent danger, regardlefs of any future bad confequences that may follow this meafure. Experience authorizes me to fay, that opium in the form of tincture, will, if in a dofe proportionate to the violence of the difeafe, give relief as fpeedily as venæfection or any other remedy.

It cannot be fufficiently regretted, that the attention of phyficians has fo feldom been directed to the effects of venæfection beyond the prefent moment. It furely is not enough, that we can with certainty prognofticate the ultimate event of a difeafe to be favourable; it is undoubtedly as much our duty, in the choice of our meafures for that purpofe, to weigh well not only their immediate, but all poffible remote confequences which may follow the ufe of them, as the future health or comfort of our patients, that neither may be endangered.

From the little that has fallen within my own obfervation, I cannot too forcibly deprecate the ufe of the lancet in croup. If we could always trace the hiftory of fuch as have, in infancy, been afflicted much with this complaint, from that period beyond which we are told that "there are no inftances of children being affected with it;" I fear that about this time, it will have been too often found, they began to be lefs active than boys ufually are at that age; that they feemed to have acquired a peculiar fufceptibility of prevailing difeafes, whether local or epidemic;

 and and not unfrequently that the early ftages of manhood were embittered by repeated attacks of Afthma.

I do not mean to contend that fuch a train of evils is the unavoidable confequence of croup in infancy, or of the method of curing it; I barely hint my fufpicions of this being too often the cafe, from knowing fuch to have been the fate of individuals of my own acquaintance, none of whom are yet beyond their thirtieth year; and may, perhaps, juftify the caution which we have ventured to give, as to the ufe of venæfection in croup, in which difeafe my practice, however, has been but fmall.

With regard to blood-letting in general, as a means of cure in inflammation, fynocha, &c. let me afk, whence the neceffity of diminifhing the quantity of blood in fuch difeafes? or what proof have we, that the quantity of blood being increafed, allowing, however, that it actually is fo, is this increafe of it the caufe of the evil? By taking blood away we undoubtedly leffen the quantity of it, but do we really diminifh the bulk of the circulating fluids, and contract the fize of the blood veffels? This is but doubtful, for it is more than probable, that from the lofs of blood the fecretions are diminifhed, and abforption of moifture* from the atmofphere increafed. From the way in which authors fo repeatedly inculcate the emptying the blood veffels as a neceffary meafure in the cure of difeafes, one would imagine they were fpeaking of them as mere elaftic tubes that dilate or contract, in proportion to the quantity of fluid impelled into them, as if they were ftimulated into action folely from the quantity of fluid which they contain, independent of the peculiar principle which the blood is known to poffefs, or their own irritability. The effects which we fee blood-letting fo inftantaneoufly produce, cannot be explained from the quantity, but the manner in which it is taken away. It may admit of a doubt whether in fynocha, the contractile power of an artery, in propelling the blood, is greater than in health: What are our proofs of its being fo?

The acutenefs of the pains in phlegmafiæ, feem to arife rather from the fpafm on the extremities of the arteries, than from

* The exceffive fweatings that often attend incipient recovery from fever, efpecially if the patient has been much exhaufted by it, as they do not feem to have a debilitating effect, are probably owing to increafed cuticular abforption, and a retrograde motion of the abforbents.

That the diarrhœa, which alfo often accompanies great debility, is occafioned by cuticular abforption, and a retrograde motion of the inteftinal abforbents, we judge from this, that we have generally been able to check it, even when exceffive, by anointing the whole body at bed time with mutton fuet, or hog's lard, which, when omitted, before the patient had acquired a fufficient degree of ftrength, the diarrhœa often returned.

from the dilation of them, or an increafed quantity of blood. Exclufively of this, viz. the fpafm of the extreme arteries, "as fupporting an increafed action of the heart and arteries," the blood itfelf feems to have acquired a new, or a higher degree of its ftimulant quality, (perhaps hyperoxygenation) whence the increafed irritability, and heat of the whole body. All which, from the known fedative* powers of opium, naturally point it out as a remedy adapted to the nature of fuch difeafes as are accompanied with fynocha, &c. If it is hurtful, how are its ill effects to be explained?

I am well aware with what caution, notwithftanding every fact that can be inftanced, to prove the beneficial employment of opium in phlegmafiæ, it will be admitted as a fafe practice. If, however, upon a full and candid trial of its ufe, as recommended, and which can be made, and its degree of credit fairly afcertained in hofpitals, this method of curing inflammatory fevers fhall receive the fanction of men juftly refpected for their great profeffional talents; this will have more influence in determining the public opinion in its favour, and of courfe bringing it into repute, than either its own intrinfic value, or all the arguments and facts that can be adduced in its favour by an obfcure individual.†

The evils which we have already alleged to arife from the injudicious, or too free ufe of the lancet, though of fufficient importance to merit an inquiry how they may be prevented, are, however, lefs ferious than thofe which we fhall next notice.

From the prevalence of bleeding in inflammatory difeafes, fome have, either from prejudice in its favour, or from a want of proper difcrimination, ufed it copioufly in genuine Typhus, accompanied (as it fometimes is) with thoracic pains, &c. The refult of fuch practice will be obvious. Of this, I faw a mournful inftance, not many months ago; and I have heard of other well attefted cafes of the fame kind, all of which proved equally fatal.

---

* That I may not afford to the Brunonians an opportunity of cavilling with me about the word fedative, as applied to opium, as this ufed formerly to be a favourite topic of controverfy among them, I fhall obferve, that Dr. Trotter's explanation of its qualities is, in my opinion, fully fatisfactory—"We no longer contend for a fedative power in this medicine (opium) than as a ftimulant exhaufting the fenforial power."—Medicina Nautica, vol. i.

† It is doubtful, if the cow-pox would have been fo favourably received by the public, or its progrefs have been fo rapid, if the fubject had not been fo induftrioufly inveftigated, and its advantages pointed out, by Drs. Jenner, Pearfon, Woodville, &c. or fome others of equal refpectability.

Besides the case of fatal blood-letting, instanced by Dr. Vaughan, in which the death of the patient is so satisfactorily accounted for by Mr. Pulley, Dr. Monro, if I recollect rightly, annually shews to his pupils the arms of three or four patients who lost their lives in a similar manner.*

As venæsection has, till lately, been a very fashionable remedy in injuries done to the head, of every kind, I do strongly suspect, that by the indiscriminate use of this evacuation in such cases of violence, in that species of injury particularly called concussion, many lives have been lost. Of this, it is satisfactory to learn, that surgeons seem at length to be convinced, and have now adopted in such cases, a very opposite method of cure indeed, and with manifest advantage.†

Now, if venæsection was in like manner to be discouraged in other kinds of disease, in which it has been found that the use of it may with advantage be superseded, and that done by authority sufficiently respectable to influence the public mind, might we not expect that equal advantages would accrue from the change?

Upon the whole, I think that I am sufficiently warranted, from experience, to draw the following conclusions respecting the use of venæsection in the practice of medicine, viz. That it is never necessary, seldom safe, often hurtful, and sometimes fatal.

The blood has justly been styled the pabulum vitæ; and so absolutely necessary is it for the preservation of a due degree of health

* It is not a little surprising how often we hear opinions and discoveries ascribed, exclusively, to the genius and industry of Mr. John Hunter. I allude to Mr. Pulley's assertion, that the disease of which Dr. Vaughan's patient died, was unknown, till Mr. Hunter taught us the nature of it. I have also to notice, an expression of Dr. Drake's, "It has *lately* been maintained, by the most celebrated Physiologists, among whom, *Mr. Hunter stands foremost*, that pus is a secreted fluid; as if Mr. Hunter was the first who proposed this theory of the formation of pus; or, that his authority was of sufficient importance to fix the public opinion. If these gentlemen have received any part of their education at Edinburgh, they must recollect to have heard Dr. Monro describe the disease abovementioned with an accuracy that bespoke his thorough knowledge of its nature; as also the morbid appearances on the arms of the unfortunate patients shown to his pupils.

With regard to pus, Dr. Monro's words are, "When an abscess is forming, you may suppose another gland added to the body." We further learn from the late Dr. Gregory's text-book, that he considered pus to be a secreted fluid. Besides, in one of the volumes of the Medical Commentaries, (that, I believe, for 1788 or 89,) there is a quotation from a book published fifty years ago by Dr. Simpson, of St. Andrews, expressive of a similar opinion as to the nature of pus.

† Discourses on the Nature and Cure of Wounds, by J. Bell, page 139.

health and vigour, both of mind and body, that, as every circumſtance of blood-letting evidently ſhews, by taking from the body this important fluid, either copiouſly or ſuddenly, we deprive it of a "ſomewhat" that, probably, is never renewed. Or there is ſome ſudden derangement of the minute organization of the body, that time cannot, or but imperfectly, repair. Though the conſtitution may from circumſtances, ſuch as loſing its blood in a ſlow and gradual manner, or from loſing it in ſmall quantities, and at ſhort intervals, be able to conform itſelf to this reduced quantity with a tolerable ſhare of health, yet that degree of vigour, which required the full quantity of blood for its ſupport, is for ever gone.

Laſtly, as all the phenomena of inflammatory fevers evidently point out their ſeat to be in the ſentient ſyſtem, and which operating as the cauſe of the irregularity of the ſanguiferous, would not our practice be more rational, if it conſiſted in the uſe of ſuch means, as from their known powers will moſt effectually, as well as ſpeedily, exhauſt the ſenſorial power, which had been accumulated during the cold ſtage, previous to the acceſſion of the pulſe, increaſed heat, local pains, &c. inſtead of then ſuddenly diminiſhing, by a large evacuation of blood, the activity of the whole body; on the entire degree of which neceſſarily depends the ſecretion or production of this ſenſorial power in ſufficient quantity for the purpoſes of life? But it may juſtly be apprehended, that the due production of ſenſorial power in quantity proportioned to the neceſſary expenditure thereof by the uſual ſtimuli, thus ſuddenly interrupted, may never again be acquired. I am, GENTLEMEN,

Your humble ſervant,

A. HUGGAN.

